# Identification of Single Nucleotide Polymorphisms and analysis of Linkage Disequilibrium in sunflower elite inbred lines using the candidate gene approach

**DOI:** 10.1186/1471-2229-8-7

**Published:** 2008-01-23

**Authors:** Corina M Fusari, Verónica V Lia, H Esteban Hopp, Ruth A Heinz, Norma B Paniego

**Affiliations:** 1 Instituto Nacional de Tecnología Agropecuaria (INTA), Instituto de Biotecnología (CNIA), CC 25, Castelar (B1712WAA), Buenos Aires, Argentina; 2 Facultad de Ciencias Exactas y Naturales, Universidad de Buenos Aires, Buenos Aires, Argentina

## Abstract

**Background:**

Association analysis is a powerful tool to identify gene loci that may contribute to phenotypic variation. This includes the estimation of nucleotide diversity, the assessment of linkage disequilibrium structure (LD) and the evaluation of selection processes. Trait mapping by allele association requires a high-density map, which could be obtained by the addition of Single Nucleotide Polymorphisms (SNPs) and short insertion and/or deletions (indels) to SSR and AFLP genetic maps. Nucleotide diversity analysis of randomly selected candidate regions is a promising approach for the success of association analysis and fine mapping in the sunflower genome. Moreover, knowledge of the distance over which LD persists, in agronomically meaningful sunflower accessions, is important to establish the density of markers and the experimental design for association analysis.

**Results:**

A set of 28 candidate genes related to biotic and abiotic stresses were studied in 19 sunflower inbred lines. A total of 14,348 bp of sequence alignment was analyzed per individual. In average, 1 SNP was found per 69 nucleotides and 38 indels were identified in the complete data set. The mean nucleotide polymorphism was moderate (θ = 0.0056), as expected for inbred materials. The number of haplotypes per region ranged from 1 to 9 (mean = 3.54 ± 1.88). Model-based population structure analysis allowed detection of admixed individuals within the set of accessions examined. Two putative gene pools were identified (G1 and G2), with a large proportion of the inbred lines being assigned to one of them (G1). Consistent with the absence of population sub-structuring, LD for G1 decayed more rapidly (r^2 ^= 0.48 at 643 bp; trend line, pooled data) than the LD trend line for the entire set of 19 individuals (r^2 ^= 0.64 for the same distance).

**Conclusion:**

Knowledge about the patterns of diversity and the genetic relationships between breeding materials could be an invaluable aid in crop improvement strategies. The relatively high frequency of SNPs within the elite inbred lines studied here, along with the predicted extent of LD over distances of 100 kbp (r^2^~0.1) suggest that high resolution association mapping in sunflower could be achieved with marker densities lower than those usually reported in the literature.

## Background

Association genetics via LD mapping is an emerging field of genetic mapping that has the potential to reach resolution to the level of individual genes (alleles) underlying quantitative traits. A Single Nucleotide Polymorphism (SNP) is a unique nucleotide base difference between two DNA sequences. In theory, SNP variations could involve four different nucleotides at a particular site, but actually only two of these four possibilities are mostly observed. Thus, in practice, SNPs are biallelic markers, so the information content on a single SNP is limited compared to the polyallelic SSR markers [1-3]. This disadvantage is overcome by the relatively larger abundance and stability of SNP loci compared to SSR loci. For instance, the usual frequency of SNPs reported for plant genomes is about 1 SNP every 100–300 bp [4]. The abundance, ubiquity and interspersed nature of SNPs together with the potential of automatic high-throughput analysis make them ideal candidates as molecular markers for construction of high-density genetic maps, QTL fine mapping, marker-assisted plant breeding and genetic association studies [5,6]. In addition, SNPs located in known genes provide a fast alternative to analyze the fate of agronomically important alleles in breeding populations, thus providing functional markers.

Several methodologies have been used to identify DNA variants [7], but usually SNPs discovery is achieved by electronic screening of comprehensive EST collections and re-sequencing of selected candidate regions from multiple or representative individuals of a target population [8-16]. Massive methods like high-density oligonucleotide probe arrays have recently emerged to identify single feature polymorphisms (SFPs) as attractive alternatives to SNPs [17]. In the last years, a number of large-scale SNP discovery projects have been carried out in crop plants to apply association analysis to crop genetic improvement [18-22]. Association analysis includes the estimation of nucleotide diversity, the assessment of linkage disequilibrium structure (LD) and/or the correlation between polymorphisms and the evaluation of selection processes. Association studies based on LD come from well-studied model species such as *Arabidopsis thaliana*, maize, rice and barley [20,21,23-27] as well as woody plants [28,29], ryegrass [30-33] and economically important crops such as wheat, soybean, sorghum and potato [34-37]. The rationale behind this approach is that nucleotide diversity not only reflects the history of selection, migration, recombination and mating systems of a given organism, but also provides information on the source of most of the phenotypic variation [38]. Systematic searches of associations between individual SNPs, or SNP haplotypes and phenotypes of interest within a suitable population would render the identification of causative variants (quantitative trait nucleotides, QTNs), leading to "gene-assisted-selection", where advantageous genotypes could be selected based on their DNA sequence reducing the costs of phenotypic testing.

Analyses of genetic diversity in sunflower (*Helianthus annuus*) were based, until very recently, solely on traditional techniques such as allozymes [39] and SSRs [40-42]. Trait mapping by allele association requires a high-density map, which could be obtained by the addition of SNPs to the SSR genetic maps already generated [43-45]. To date, the only data available on sunflower nucleotide diversity comes from the study of 9 genomic loci in 32 wild populations and exotic germplasm accessions [46] and of 81 RFLP loci in 10 inbred lines [47]. However, further investigation of the nature, frequency and distribution of sequence variation is still needed to better understand the range of diversity and the origin of the genetic changes associated with domestication and agronomic improvement. Indeed, the choice of germplasm is crucial for the discovery of useful alleles, and a genotypically diverse set of germplasm must be chosen to achieve this goal. Furthermore, the inclusion of candidate regions putatively related to biotic or abiotic stresses might help zeroing in on candidate tagged SNPs to evaluate allele association in sunflower germplasm.

Here, we present a survey of nucleotide diversity at 28 loci related to biotic and abiotic stresses from 19 sunflower public elite inbred lines that are well recognized breeding materials representing the species diversity [42,48-50]. The aims of this study were to: (1) determine the frequency and the nature of the SNPs and indels in current breeding populations, (2) examine the effects of population structure on LD assessment, (3) compare the resulting nucleotide diversity and LD estimates to those previously reported for wild and cultivated sunflower.

## Results

### SNPs frequency and nucleotide diversity

A total of 64 candidate regions related to biotic and abiotic stresses were selected for SNP identification and nucleotide diversity analyses (Additional file 1). Single PCR products of the expected sizes were detected for 40 regions (62.50%) and 28 of them (43.75%) yielded high-quality sequence data. The features and polymorphism indices of the 28 candidate genes used for subsequent analyses are shown in Table 1 [GeneBank Acc. Nos. EU112474–EU112815, EU112835–EU113005, EU113025–EU113043]. The 28 genomic loci were amplified in 19 genotypes representative of cultivated sunflower germplasm, comprising 14,348 bp of aligned sequence per individual. Each gene alignment ranged from 100 to 1,114 bp including indels. Further inspection of Table 1 reveals the occurrence of at least 1 SNP in 24 out of 28 genes evaluated, with a total of 207 nucleotide changes identified among all genes and individuals analyzed. Thus, an average of 1 SNP every 69 bp (excluding indels) and a mean number of 7.39 SNPs per region were detected. As expected, occurrence of synonymous substitutions (85) was fourfold larger than non-synonymous SNPs (20) and 70.53% of transitions were found. The number of SNPs varied also between coding and non-coding regions: 105 SNPs were found in 9,506 bp of coding regions whereas 102 SNPs were detected in 4,842 bp of intergenic or intragenic non-coding sequences: hence, the SNP frequency was 1 SNP/90 bp in coding regions and 1 SNP/48 bp in non-coding regions. These results suggest that coding regions are more conserved (less SNP frequency) than non-coding regions, most probably due to purifying selection. On the other hand, the number of indels varied across genes from 0 to 11, counting 38 indel polymorphisms in the complete data set. The frequency found for indels was 1/377.6 bp reaching an average of 1.36 indels per region analyzed. Indel sizes were highly variable, ranging from a single nucleotide to 52 bp in CAM (Table 1). In some instances, the precise number of insertion and/or deletion events giving rise to each indel block was difficult to establish, especially in those regions where variable numbers of base pairs were added or deleted in different individuals in the same block. Interestingly, 3 indels were found in coding regions: 2 in the MADSB-TF3 (3 bp) and 1 in GADPH (1 bp). All indels were excluded from subsequent analyses except for both haplotype and haplotype diversity analyses in GO, LZP, GLP and GPX candidate regions (see also Table 2).

**Table 1:** Genes ID, analyzed length and total polymorphisms found in 19 sunflower inbred lines

**Strategy of selection**	**Gene**	**Similarity (BLASTx searches)^a^**	**Description**	**S_T_^b^**	**N° Indels^c^**	**Total length (bp)^d^**	**Coding region (bp)^d^**	**Noncoding region (bp)^d^**
**Sunflower SSH-EST library survey**	**GO**	Glycolate oxidase (*Spinacia oleracea*)	Electron carrier ROS machinery [69]	2	1 (36)	608	300	308
	**PGIP3**	Poligalacturonase inhibitor protein precursor (*Actidinia deliciosa*)	Plant defense against diverse pathogens that use polygalacturonase to breach the plant cell wall [70]	3	0 (0)	676	561	115
	**LZP**	Leucine zipper protein putative (*Triticum aestivum*)	Transcriptional factors involved in plant development, photomorphogenesis and responses to stress [71]	0	1 (8)	425	84	341
	**GLP**	Germin-like protein (*Oryza sativa*)	Apoplastic and glycosilated protein shown to be involved in plant defense [72]	0	3 (3)	876	648	228
**Literature search**	**MADSB-TF3**	MADS-box transcription factor (*Helianthus annuus*)	Transcription factors acting as regulators of various aspects of plant development [73]	13	11 (20)	1082	291	791
	**AALP**	*Arabidopsis *Aleurain-like protease (*Arabidopsis thaliana*)	Enzyme involved in macromolecular degradation and recycling, its expression is up-regulated during aging-related and harvesting-induced senescence [74]	10	2 (11)	269	189	80
	**LIM**	LIM domain protein PLIM1b (*H. annuus*)	Transcription factors that play important roles in construction of cytoskeleton and signal transduction [75]	6	2 (5)	319	150	169
***in silico *analysis with SNP Discovery**	**RL41**	60S ribosomal protein L41 (*A. thaliana*)	Protein component of the Ribosomal 60S subunit, important for the translational apparatus and involved in apoptosis and cell cycle [76, 77]	3	0 (0)	100	66	34
	**ANT**	Adenine nucleotide translocator, mitochondrial precursor (*Gossypium hirsutum*)	Inner-membrane mitochondria carrier that plays roles in integrating celullar stress and regulating programmed cell death [78]	9	0 (0)	216	213	3
	**RS16**	40S ribosomal protein S16 (*Euphorbia esula*)	Ribosomal S16 component retained during desiccation process in water stress tolerant plants [79]	7	0 (0)	448	405	43
	**NsLTP**	Nonspecific lipid-transfer protein precursor (*H. annuus*)	Participates in cutin formation, embryogenesis, defense reactions against phytopathogens, symbiosis and adaptation to various environmental conditions [80]	7	2 (13)	294	96	198
	**SEM**	Probable 26S proteasome complex subunit sem1–2 (*H. annuus*)	Complex involved in protein turnover pathway, helps to remove proteins that arise from synthetic errors, spontaneous denaturation, free-radical and enviromental stress induced damage [81]	3	0 (0)	226	87	139
	**SAMC**	S-adenosylmethionine decarboxylase (*Daucus carota*)	Key enzyme in PolyAmines (PAs) biosynthesis. PA synthesis is induced by high osmotic pressure, low temperature, low pH and oxidative stress. PAs are proposed to have resistance roles in plant-microbe interactions [82]	12	1 (3)	369	189	180
	**GCvT**	Glycine cleavage symstem T protein (*Flaveria trinervia*)	The glycine cleavage system catalyzes the oxidative decarboxylation of glycine in bacteria and in mitochondria of animals and plants [83]	3	0 (0)	183	180	3
	**SBP**	Sedoheptulose-1,7-bisphosphatase, chloroplast (*A. thaliana*)	Calvin Cycle's enzyme: branch point between regeneration of ribulose 1,5 biphosphate and export to starch biosynthesis. The overexpression of SBP increases photosynthetic carbon fixation and biomass in plants [84]	11	0 (0)	243	240	3
	**LHCP**	Light-harvesting chlorophyll a/b-binding protein precursor (*L. sativa*)		8	0 (0)	362	348	14
	**CPSI**	Photosystem I reaction center subunit V, chloroplast precursor (*Camellia sinensis*)	Genes encoding components involved in photosynthesis which showed differential expression patterns under both chilling and salt stresses in sunflower [69]	4	0 (0)	168	144	24
	**PSI-III-CAB**	Pothosystem I type III chlorophyll a/b-binding protein (*A. thaliana*)		1	1 (1)	710	387	323
	**CAB**	Chlorophyll a/b-binding protein (*Beta vulgaris*)		7	2 (10)	537	393	144
**Comparison purposes**	**CAM**	Calmodulin (*Morus nigra*)	Plays a central role in calcium-mediated signaling [46]	29	6 (93)	538	117	421
	**CHS**	Chalcone synthase (*Saussurea medusa*)	Plays an essential role in the biosynthesis of plant phenylpropanoids [46] and abiotic stress defense responses [85, 86]	0	0 (0)	1051	978	73
	**GAPDH**	Glyceraldehyde-3-phosphate dehydrogenase (*Glycine max*)	Tetrameric NAD1 binding protein that is involved in glycolysis and gluconeogenesis [46]	2	2 (3)	782	617	165
	**GIA**	Gibbelleric acid insennsitive-like protein (*Lactuca. sativa*)	Putative gibberellin response modulator [46]	2	1 (1)	749	504	245
	**GPX**	Putative gluthathione peroxidase (*Medicago truncatula*)	Antioxidant enzymes suggested as important factors in protection mechanisms against oxidative damage [46]	0	1 (6)	744	513	231
	**GST**	Glutathione S-transferase (*Pisum sativum*)		40	0 (0)	561	351	210
	**PGIC**	Cytosolic phosphoglucose isomerase (*Stephanomeria tenuifolia*)	Catalyzes the reversible isomerization of 6-phosphoglucose and 6-phosphofructose, an essential reaction that precedes sucrose biosynthesis [46]	15	2 (4)	569	219	350
	**SCR1**	Scarecrow transcription factor type 1(*Castanea sativa*)	SCARECROW-like gene regulators are known to be involved in asymmetric cell division in plants [46]	3	0 (0)	739	732	7
	**SCR2**	Scarecrow transcription factor type 2 (*O. sativa*)		7	0 (0)	504	504	0

**Total**				**207**	**38 (217)**	**14,348**	**9,506**	**4,842**

**Average/locus**				**7.39**	**1.36**			

**Frequency**				**1/69**	**1/377.6**			

^a^Gene coding regions and functions were determined by BLASTx searches.

^b^Total single nucleotide polymorphisms (S_T_).

^c^Number of indels counted according to blocks of insertions and deletions. The total bp length of indels is displayed in brackets.

^d^Total length, coding and non-coding region are displayed excluding indels.

**Table 2:** Measures of nucleotide diversity and Tajima's D

**Gene**	**S_I_^a^**	**θ_w_**	**π_T_**	**π_sil_**	**π_syn_**	**π_nonosyn_**	**π_nonsyn_/π_syn_**	**N° haplotypes**	**Haplotype diversity**	**Tajima's D**
**GO**	0	0.0009	0.0004	0.0003	0	0.0005	-	3	0.205	-1.51
**PGIP3**	3	0.0013	0.0018	0.0050	0.0062	0	0	4	0.725	1.10
**LZP**	0	0	0	0	0	0	-	2	0.281^b^	-
**GLP**	0	0	0	0	0	0	-	3	0.433^b^	-
**MADSB-TF3**	5	0.0034	0.0025	0.0027	0.0159	0.0018	0.1141	9	0.801	-1.02
**AALP**	6	0.0119	0.0117	0.0203	0.0143	0	0	4	0.661	-0.08
**LIM**	5	0.0056	0.0076	0.0117	0.0092	0	0	4	0.579	1.13
**RL41**	1	0.0087	0.0071	0	0	0.0145	-	3	0.556	-0.50
**ANT**	9	0.0122	0.0225	0.0841	0.0888	0	0	2	0.526	2.93^***c^
**RS16**	5	0.0047	0.0066	0.0206	0.0294	0	0	3	0.573	1.36
**NsLTP**	5	0.0068	0.0077	0.0084	0.0380	0.0057	0.1504	3	0.433	0.42
**SEM**	1	0.0038	0.0018	0.0027	0	0	-	3	0.205	-1.42
**SAMC**	7	0.0093	0.0084	0.0134	0.0357	0.0007	0.0204	5	0.684	-0.34
**GCvT**	2	0.0047	0.0064	0.0253	0.0270	0	0	3	0.579	0.95
**SBP**	6	0.0142	0.0137	0.0523	0.0550	0	0	5	0.760	-0.14
**LHCP**	8	0.0063	0.0079	0.0268	0.0313	0.0011	0.0341	3	0.602	0.8266
**CPSI**	2	0.0068	0.0041	0.0101	0.0059	0.0010	0.1616	2	0.298	-1.17
**PSI-III-CAB**	1	0.0004	0.0006	0.0010	0	0	-	2	0.409	0.79
**CAB**	7	0.0038	0.0059	0.0136	0.0203	0	0	3	0.485	1.91
**CAM**	18	0.0155	0.0137	0.0166	0.0217	0	0	6	0.801	-0.44
**CHS**	0	0	0	0	0	0	-	1	0.000	-
**GAPDH**	1	0.0008	0.0007	0.0017	0	0	-	3	0.485	-0.24
**GIA**	2	0.0008	0.0005	0.0011	0.0016	0	0	2	0.199	-0.73
**GPX**	0	0	0	0	0	0	-	2	0.256^b^	-
**GST**	31	0.0204	0.0277	0.0464	0.0636	0.0080	0.1254	9	0.772	1.44
**PGIC**	13	0.0081	0.0055	0.0074	0.0021	0.0012	0.5673	4	0.298	-1.19
**SCR1**	3	0.0012	0.0018	0.0076	0.0079	0	0	3	0.649	1.39
**SCR2**	7	0.0040	0.0037	0.0126	0.0126	0.0009	0.0721	3	0.374	-0.26

**Average**	**5.29**	**0.0056**	**0.0061**	**0.0140**	**0.0174**	**0.0013**	**0.0655**	**3.54**	**0.497**	

^a^Parsimony informative sites (S_I_) used to measure nucleotide diversity.

^b^The number of haplotypes and haplotype diversity values was obtained by using indel polymorphisms.

^c^Tajima's D significant p < 0.001.

Summarizing, moderate levels of DNA polymorphism were found (Table 2). Genetic variation at the nucleotide level was estimated from mean nucleotide diversity (π_T _= 0.0061) and from the number of segregating sites (θ_W _= 0.0056). Average silent-site diversity (π_sil _= 0.0140) and synonymous-site diversity (π_syn _= 0.0174) were higher than non-synonymous changes (π_nonsyn _= 0.0013). In 26/28 loci examined, π_nonsyn _was either 0 or lower than π_syn_, suggesting that the diversity of these regions is governed by purifying selection. However, the GO and the RL41 regions showed π_nonsyn _higher than π_syn_. In GO π_nonsyn _was 0.00047, while π_syn _was 0; a single nucleotide substitution in the RHA293 inbred line, is responsible for this difference. In RL41 the non-synonymous substitutions are caused by 2 singletons present in HA292 and by a parsimony informative site which separates HA61, HA89, HA303, KLM280, PAC2, RHA266 and RHA274 from the remaining inbred lines. This substitution is a C/A transversion in the 2^nd ^codon position and causes the change from a Proline to a Glutamine (i.e. a change from a non-polar to a polar aminoacid). Whether this site is essential for the protein to be functional still remains to be determined. Despite the fact that SNP frequency was higher in non-coding than in coding regions, the average nucleotide polymorphism and nucleotide diversity of non-coding regions (θ_W _= 0.0052, π_T _= 0.0053) was only slightly higher, although non-significant, than diversity estimates in coding regions (θ_W _= 0.0047, π_T _= 0.0053).

The number of haplotypes per locus ranged from 1 to 9 among the 19 inbred lines and average haplotype diversity was 0.497. Although LZP, GLP and GPX sequences did not display any SNP polymorphism, the indels exhibited in these candidate genes were enough to determine distinct haplotypes, with haplotype diversity values of 0.281 (LZP), 0.433 (GLP) and 0.256 (GPX).

In terms of allele frequency distribution, even though Tajima's D was not significantly different from 0 in 27/28 regions (Table 2), it was significantly positive in ANT (D = 2.93, p < 0.001). Positive Tajima's D value indicates a deficit of low frequency alleles relative to neutral expectations in a randomly mating population of constant size. In this context, positive D values could be the consequence of population bottlenecks, population subdivision or balancing selection as would be expected in breeding populations.

To avoid the distortions introduced by gene sampling, the estimates of diversity were recalculated for the 19 inbred lines included in this work and for the Primitive and Improved accessions (P&I) chosen by Liu and Burke [46] using only the subset of genes in common for both studies (Table 3). The θ_W _average values were 0.0056 for the 19 inbred lines, 0.0078 for the P&I cultivated group and 0.0079 for the pooled accessions. In addition, the π_T _values were 0.0060, 0.0057, and 0.0069, respectively. Therefore, the nucleotide diversity estimates (θ_W _and π_T_) for the 19 inbred lines analyzed in this work remained the same regardless of the loci being surveyed.

**Table 3:** Evaluation of gene sampling effects on diversity estimates.

		**Genes analyzed**									**MEAN from 9 genes**	**MEAN from all regions**
**Parameters**	**Group of germplasm**	**CAM**	**CHS**	**GAPDH**	**GIA**	**GPX**	**GST**	**PGIC**	**SCR1**	**SCR2**		
**θ_W_**	19 inbred lines	0.0155	0	0.0008	0.0008	0	0.0204	0.0081	0.0012	0.0040	**0.0056**	**0.0056^a^**
	Improved and Primitive	0.0176	0.0005	0.0006	0.0013	0.0047	0.0190	0.0157	0.0051	0.0054	**0.0078**	**0.0072^b^**
	All accessions pooled	0.0175	0.0004	0.0006	0.0015	0.0043	0.0222	0.0145	0.0046	0.0053	**0.0079**	**-**

**π_T_**	19 inbred lines	0.0137	0	0.0007	0.0005	0	0.0277	0.0055	0.0018	0.0037	**0.0060**	**0.0061^a^**
	Improved and Primitive	0.0138	0.0003	0.0011	0.0008	0.0021	0.0124	0.0109	0.0060	0.0042	**0.0057**	**0.0056^b^**
	All accessions pooled	0.0144	0.0002	0.0010	0.0007	0.0014	0.0262	0.0090	0.0051	0.0040	**0.0069**	**-**

The 9 regions (CAM, CHS, GAPDH, GIA, GPX, GST, PGIC, SCR1 and SCR2) in common with Liu and Burke report were re-analyzed in the inbred lines (19 alleles/19 accessions), the improved and primitive cultivated accessions surveyed by Liu and Burke (32 alleles/16 accessions) [46] and the complete set of accessions pooled together (51 alleles). The diversity estimates (π_T _and θ_W_) displayed the same pattern independently the loci surveyed.

^a^Nucleotide polymorphism and nucleotide diversity obtained with the complete set of 28 genes studied in Table 2.

^b^ Nucleotide polymorphism and nucleotide diversity obtained by Liu and Burke [46]

### Linkage disequilibrium (LD)

**Figure 1:**
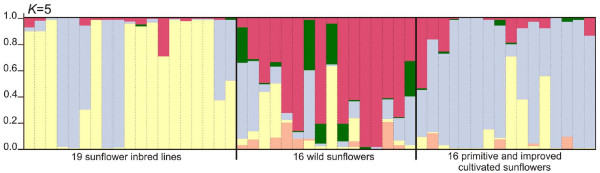
**Population structure in sunflower inbred lines**. Dash lines separate each individual, which is partitioned in K coloured segments that represent the individual's estimated membership fractions in K clusters. Black lines separate individuals from different groups. First group is composed by the 19 sunflower inbred lines (in order from left to right: HA52, HA61, HA89, HA292, HA303, HA369, HA370, HA821, HAR2, HAR3, HAR5, KLM280, PAC2, RHA266, RHA274, RHA293, RHA374, RHA801 and V94); the second and the third group are the individuals studied by Liu and Burke [46]. The inbred-lines group has mostly contributions of two clusters (yellow and light-blue).

**Figure 2:**
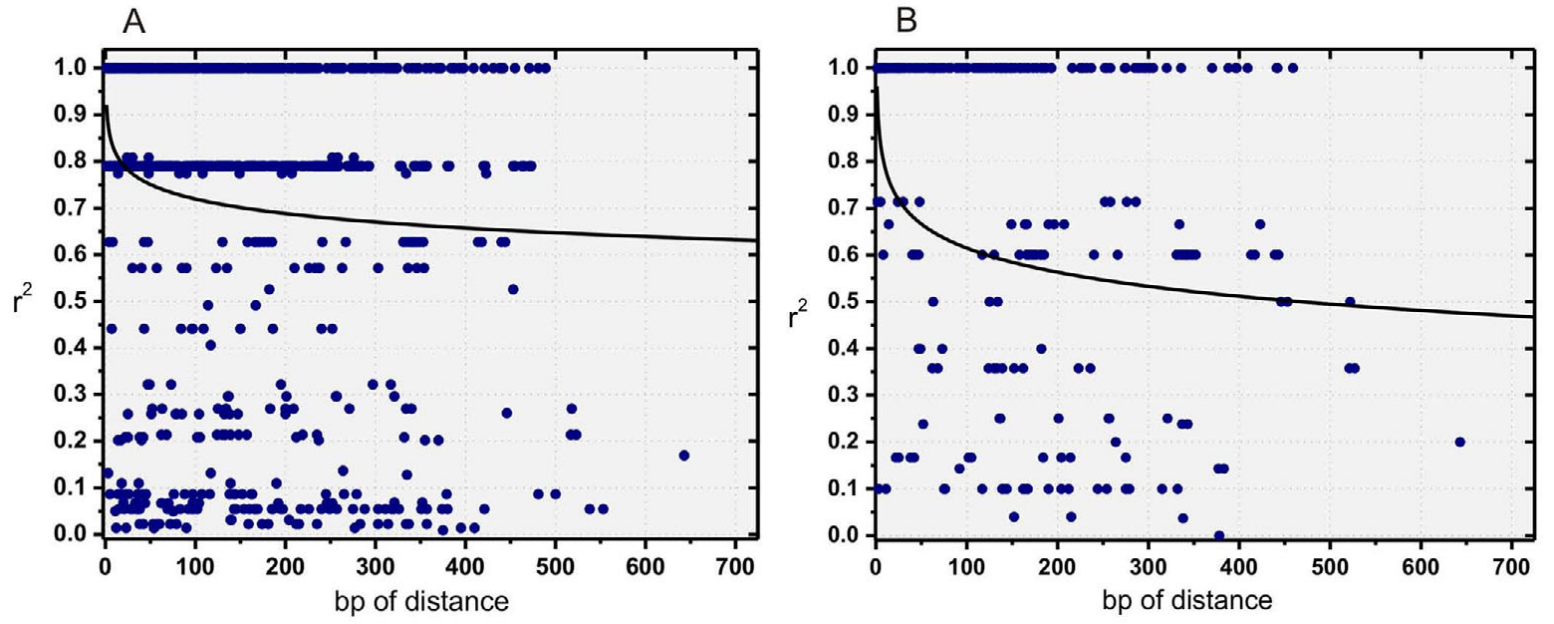
**Linkage disequilibrium**. **A**: LD plot from 24 genes pooled together for the 19 inbred lines. The logarithmic trend line reaches a value of 0.64 at 643 bp. **B**: LD plot from the whole gene data calculated for the G1 subset of individuals identified in the STRUCTURE analysis (HA52, HA61, HA89, HA370, HAR3, HAR5, KLM280, PAC2, RHA266, RHA274, RHA293 and RHA374).

The presence of population structure can lead to spurious results and must be considered in the statistical analysis [51]. Therefore, as a preliminary step to the assessment of LD, population structure was analyzed using the model-based approach reported by Pritchard et al. [52], employing 136 non-linked SNP loci derived from the 9 genes shared between the 19 inbred lines studied in this work and the 32 wild and cultivated individuals previously reported by Liu and Burke [46]. This test was useful to prevent spurious associations that arise for reasons other than physical proximity and to assess the real extent of LD. The highest log likelihood scores were obtained when the number of populations was set to five. Each individual's inferred ancestry to the five model-based populations is presented in Figure 1. The 19 elite accessions examined here are mainly composed by the contribution of two gene pools (yellow and light-blue, Figure 1), with most of their inferred ancestries being higher than 80%. These two gene pools are also the main constituents, but in a different proportion, of the cultivated accessions analyzed by Liu and Burke [46]. As expected, the wild accessions have a more diverse ancestry, with contributions from all five model-based populations identified. On the basis of population structure analysis, two groups can be defined within the 19 inbred lines studied in this work. The first group (G1) is composed by HA52, HA61, HA89, HA370, HAR3, HAR5, KLM280, PAC2, RHA266, HA274, RHA293 and RHA374 (yellow gene pool); the second group (G2) includes HA292, HA303, HA369, HA821, HAR2, RHA801 and V94 inbred lines (light-blue gene pool). According to the method's assumptions, these two groups are characterized by different sets of allele frequencies. For this reason, pairwise estimates of LD (i.e. r^2^) were calculated for: (i) the entire set of inbred lines (Figure 2A), and (ii) the subset of inbred lines from G1 (Figure 2B). The G2 subset was not included in this analysis because of its small number of individuals. Figure 2 displays the scatter plots of r^2 ^versus the physical distance between all pairs of SNP alleles within a gene, pooled for the 24 polymorphic regions included in this work. Since all regions are <1 kbp long this analysis reveals disequilibrium patterns at short distance. For the entire set of genotypes, the logarithmic trend line declines very slowly, reaching a value of 0.64 at 643 bp (Figure 2A). Conversely, when the LD plot includes only the genotypes belonging to G1 group, the logarithmic trend decays more rapidly and the value is 0.48 for the same distance (Figure 2B). As expected, there is clearly a bias towards higher levels of LD when the population structure in the sample is not factored into the analysis. Interlocus analyses revealed no LD between loci (data not shown).

## Discussion

### SNPs frequency and nucleotide diversity

Candidate genes were selected from SSH-EST collection, literature and *in silico *analysis attending to their putative role in biotic and/or abiotic stresses, while other randomly selected regions were included as controls. They were properly sequenced in 19 very well known inbred lines used in breeding programs and different patterns of polymorphisms were obtained. The SNP frequency detected in our set of elite accessions was 1 SNP/69 bp: whereas it is quite comparable to the frequency obtained by Ching et al. for maize inbred lines (1 SNP/60.8 bp) [24], it is higher than the frequency reported by Tenaillon et al. (1 SNP/104 bp) also for maize [53]. Nevertheless, the discrepancy between maize studies could be caused by differences in gene sampling. Moreover, the abundance of SNPs that we found in sunflower is comparable to the one described in a *Pinus taeda *report, which exhibited 1 SNP/63 bp [28]. On the other hand, other agronomically important crops like sorghum (1 SNP/123 bp) [34], soybean (1 SNP/328 and 1 SNP/536) [16,37] and rice (1 SNP/113 bp and 1 SNP/100 bp) [20,25] presented a lower SNP frequency than the sunflower inbred lines surveyed in this work.

SNP occurrence in sunflower as well as nucleotide diversity values were reported recently by Liu and Burke for 16 primitive and improved accessions (1 SNP/39 bp, θ_W _= 0.0072, π_T _= 0.0056) and by Kolkman et al. for 10 inbred lines (1 SNP/46 bp, θ_W _= 0.0094, π_T _= 0.0107) [46,47]. The differences among these values and the estimates described in this work might be explained by: (i) the expected differences in the genetic divergence of the materials analyzed (primitive and early improved germplasm accessions versus elite breeding lines), (ii) the different sources of variation being considered (e.g. indel definition) and (iii) the differences in quantity and/or selection criteria of the genomic regions sequenced. Concerning the last statement, 19 out of 28 candidate genes selected in this work were uncharacterized novel regions including putative stress related proteins as well as randomly selected loci, which represent a good collection of the genome-wide expected pattern of SNPs. To determine whether the effect of interlocus variance (gene sampling) may distort the nucleotide diversity estimates (θ_W _& π_T_), we re-analyzed the sequence data of the 9 shared genes between the 19 inbred lines surveyed in this report and the P&I accessions analyzed by Liu and Burke [46]. The mean θ_W _in the inbred lines (0.0056) still remained lower and the mean π_T _(0.0060) remained higher than the re-calculated estimates for the P&I individuals (θ_W _= 0.0078 and π_T _= 0.0057) (Table 3). These results confirm the pattern previously observed for the entire set of genes formerly analyzed in the 19 inbred lines. In addition, the θ_W _and π_T _from the 9 genes for the pooled accessions were higher than both, the 19 inbred lines and P&I individual estimates. Consequently, these discrepancies are not caused by gene sampling and therefore, they might reflect genuine differences in the levels of polymorphism for different groups of individuals. While the θ_W _is based on the number of segregating sites and is influenced by the presence of rare alleles, the π_T _is a measure of the pairwise differences between two sequences. A deficiency of rare alleles is expected under the pronounced bottlenecks that lead to the origin of inbred lines and the increased in pairwise differences can result from the divergent nature of the elite materials selected for this study. The analyses of the pooled data confirmed those differences between the sources employed in both works, thus, weighting not only the presence of rare alleles in P&I accessions, but also the divergent nature of elite inbred lines. Wild sunflowers showed SNP occurrence (1 SNP/19 bp) and nucleotide diversity values (θ_W _= 0.0144; π_T _= 0.0128) [46] higher than the estimates obtained for the 19 elite inbred lines, which is in agreement with our expectations because of the history of artificial selection, recombination and improvement of the last ones.

Regarding synonymous and non-synonymous changes, in the 19 inbred lines average silent-site diversity (π_sil _= 0.0140) and synonymous-site diversity (π_syn _= 0.0174) were higher than mean non-synonymous changes (π_nonsyn _= 0.0013), however, 2 loci showed higher π_nonsyn _than π_syn _(GO: π_nonsyn _= 0.00047 and π_syn _= 0; RL41: π_nonsyn _= 0.0145 and π_syn _= 0). Particularly in RL41, one non-synonymous substitution is a parsimony informative site that changes the protein sequence at that codon position. Nevertheless, this kind of changes are frequently seen on inbred lines that were subjected to artificial selection, for instance, missense changes were observed in invariant sites of HD proteins of rice cultivars as a probable consequence of artificial selection during the domestication process [54].

Concerning the evaluation of selection, most of the genes (27/28) showed Tajima's D values which were not significantly different from 0, while one region showed a significantly positive Tajima's D (ANT, D = 2.93; p < 0.001). As mentioned before, positive D values could be the consequence of population bottlenecks, population subdivision or balancing selection. These factors are likely to be operational in sunflower elite lines. The population bottleneck caused by inbreeding may change the rate of allelic frequency and the conditions for a stable polymorphism in the entire data set. Hence, the data presented above do no adjust to this hypothesis. In contrast, selection is the factor that might probably affect D values in only one gene. Anyway, neither population bottlenecks nor selection can be proved without a more comprehensive and genome-wide study in sunflower.

### Linkage Disequilibrium assessment

Linkage equilibrium and LD are population genetics terms used to describe the likelihood of co-occurrence of alleles at different loci in a population. Generally, linkage refers to the correlated inheritance of loci through physical connection on a chromosome [1]. Population subdivision and admixture increase LD, but their effects depend on the number of populations, the rate of exchange between populations and the recombination rate [55]. Association analysis based on LD has been employed recently in plants, with initial resistance due in large part to the confounding effects of population structure and the general lack of knowledge regarding the structure of LD in many plant species [56]. The complex breeding history of sunflower inbred lines and the consequent stratification of the germplasm may lead to an overestimation of the extent of LD, therefore extending non-random correlations to physically un-linked loci and thus making association mapping to fail. Inclusion of population structure in association models is critical for meaningful analysis [56]. The model-based clustering method of Pritchard [52] showed that inbred lines examined in this work were further sub-structured into two groups: G1 and G2 (Figure 1). LD decay was slightly slower for the entire genotype set than for the G1 group (Figure 2). Therefore, the line through the G1 data (Figure 2B) is in concordance with the LD analysis showed by Kolkman et al. [47]. Despite the short-range LD that we were able to asses, the trend line for the G1 reaches a value of 0.32 at 5500 bp, in agreement with the values obtained by Kolkman et al. [47]. The patterns of pairwise LD differed greatly between the wild sunflowers and cultivated samples analyzed here: in the former group, the strong linkage disequilibrium was evidenced within distances <200 bp [46], whereas in the second group it was noticeable at least up to 700 bp (Figure 2). The same pattern was observed in both the P&I cultivated samples analyzed by Liu and Burke [46] and in the set of inbred lines analyzed by Kolkman et al. [47]. Patterns of LD in other organisms are quite variable. For maize inbred lines [24] non-significant decay was observed in LD (r^2^) within the 600 bp analyzed, as it was found in sunflower inbred lines. However, assessments in chromosome 1 of maize landraces and inbred lines showed LD decay within 200–300 bp [53]. In addition, SNPs-LD in other maize loci and individuals evidenced a negligible level of LD (i.e.: r^2 ^< 0.1) at 1500 bp of distance [27] reflecting the rapid decay of LD in out-crossing species. *Solanum tuberosum*, despite being an out-crossing species, showed intermediate LD values (r^2 ^= 0.21 at 1 kbp; r^2 ^= 0.14 at ~70 kbp) [35] probably as a consequence of its vegetative propagation system. On the other hand, selfing species showed a larger extent of LD: >50 kbp in soybean [37], >150 kbp in *Arabidopsis *[26] and ~100 kbp in rice [25]. Similarly, LD in sorghum (high self-pollination rate), apparently dissipates within 10 kbp [34]. These last organisms seem to have LD patterns more comparable to the results presented in this work for cultivated sunflower.

## Conclusion

This study contributes to previously reported analyses of nucleotide diversity and linkage disequilibrium in sunflower [46,47]. Knowledge about genetic relationships between breeding materials could be an invaluable aid in crop improvement strategies. Analysis of genetic diversity in germplasm collections can facilitate reliable classification of accessions and identification of core accessions subsets with possible utility for specific breeding purposes. Sunflower inbred lines showed a frequency of 1 SNP per 69 bp, with nucleotide diversity estimates of θ_W _= 0.0056 and π_T _= 0.0061. As expected, these moderate levels of diversity were lower than diversity estimates found in wild accessions of sunflower [46,47]. The population structure analysis identified the subset of inbred lines that belong to a unique gene pool (G1), and helped us to assess the extent of LD without spurious associations. The extent of LD from the G1 group adjusted more accurately with previously reports of LD in cultivated sunflower [46,47] and the trend line predicted a decay of LD (i.e. r^2^~0.1) within the 100 kbp. The data presented in this work could facilitate association mapping in sunflower with lower marker densities than those usually reported in the literature for other plant species, at least at a rough scale.

## Methods

### Plant material and genomic DNA extraction

The set of 19 elite sunflower inbred lines (*Helianthus annuus *L.) selected for SNP discovery are described in Table 4. These public inbred lines represent a wide range of genetic diversity from the sunflower breeding materials as it is shown by the pedigree details. They include contributions from Russian, Canadian, Romanian and North American *H. annuus *accessions and from interspecific crossings with *H. argophyllus *and *H. petiolaris *made in Argentinean breeding programs. Particularly, they were chosen according to their morphological and agronomical characteristics regarding phenotypic behaviour against fungal pathogens, abiotic stress, seed number per capitulum and high oil yield. Among these genotypes, 15 inbred lines were previously used in the development of 550 novel microsatellites [42]. The remaining lines (HA89, RHA801, RHA266 and PAC2) are well known international reference genotypes and parental lines of well characterized mapping populations [57]. The DNA was extracted from lyophilized leaves (3-week old plants grown in greenhouse) with Nucleon™ Phytopure™ genomic DNA extraction Kit (GE, Healthcare Life Sciences, Buenos Aires, Argentina) and using previously described protocols [42].

**Table 4:** Description of the sunflower inbred lines used for SNPs and indels screening

**Inbred line**	**Pedigree**	**Location of breeding reselection**	**Features**
**H52**	Putatively Romanian germplasm^*a*^	South Africa	Oilseed maintainer
**HA61**	"953-88-3"/"Armavirski 3497"	U.S.A.	Oilseed maintainer
**HA89**	"Vniimk 8931"	U.S.A.	Oilseed maintainer
**HA292**	"Commander"*3/"Mennonite RR"^*b*^	U.S.A.	Non-oilseed maintainer
**HA303**	"Voshod"	U.S.A.	Oilseed maintainer
**HA369**	"Teguá INTA" (Arg. 8018)	Argentine	Oilseed maintainer
**HA370**	"RK-74-198"	South Africa	Oilseed maintainer
**HA821**	"HA 300" (derived from "Peredovik 301")	U.S.A.	Oilseed maintainer
**HAR2**	"Impira INTA" Selection 5	Argentine	Oilseed maintainer
**HAR3**	"Charata INTA"^*c *^selection	Argentine	Oilseed maintainer
**HAR5**	"Guayacán INTA"^*d *^selection	Argentine	Oilseed maintainer
**KLM280**	"KLM"^*e *^selection	Argentine	Oilseed maintainer
**PAC2**	*H. petiolaris *× HA61	France	Stem-head rot resistance
**RHA266**	Wild *H. annuus *× Peredovik	U.S.A	Downy mildew resistance
**RHA274**	("cmsPI343765"/"Ha119"/"Ha64-4-5")/T66006-2^*f*^	U.S.A.	Oilseed restorer
**RHA293**	"HA155"/"HIR34"/2/"RHA282"	U.S.A.	Non-oilseed restorer
**RHA374**	"Arg-R43"	U.S.A.	Oilseed restorer
**RHA801**	Multiple source R-line population	U.S.A	Fertility restorer line
**V94^*g*^**	"Mp543"* h./H. Argophyllus	Argentine	Oilseed maintainer

^*a*^"HA52" is an accession putatively originating from Romanian germplasm bred in Potchestfrom, Transvaal, South Africa.

^*b*^Third generation backcross of "Mennonite RR" to "Commander".

^*c*^"Charata INTA" was obtained by interspecific crossings with wild germplasm belonging to species *H. annuus *subsp. *annuus *and *H. petiolaris*.

^*d*^"Guayacán INTA" derived from a cross between the Argentine variety Klein and "CM953-102" and backcrossed once again with "Klein".

^*e*^"KLM" is a multiple cross between cultivars Klein × Local (a pool of local varieties of INTA Pergamino breeding program including "Guayacán INTA", "Charata INTA") × "Manfredi" (a pool of varieties from INTA Manfredi breeding program including "Impira INTA", "Cordobés INTA", "Manfredi INTA").

^*f*^T66006-2 comes from Peredovik*2/953-102-1-1-41.

^*g*^"V94" is another Argentine selection of a cross between cultivated sunflower ("MP543") and wild species (*H. argophyllus*), "MP543" derives from "MPRR" (mezcla precoz resistente a roya: pool of early material resistant to sunflower rust), which also derives from wide crossings with *Helianthus *wild species.

### Selection of candidate regions

Additional file 1 displays the 64 candidate regions selected for SNP identification, the accession numbers of the sequences used for primer design and the putative functions associated by BLASTx searches, together with the protein accession best hit. The 62.50% (40 regions) were amplified in 2 genotypes in a preliminary test, while 43.75% (28) yielded high-quality sequence data for the entire set of genotypes. The IDs of the 28 candidate genes used for subsequent analyses are outlined in Table 1. Briefly, four candidate genes, Glicolate Oxidase (GO, EC 1.1.3.15), Poligalacturonase Inhibitor Protein Precursor (PGIP3), Leucine Zipper Protein (LZP) and the Germin-Like Protein (GLP, which is a putative Oxalate Oxidase, EC 1.2.3.4) were chosen from a SSH-EST collection [58] since they are putatively involved in sunflower biotic and abiotic stress resistance mechanisms. The MADS-Box Transcription Factor (MADSB-TF3) and the two senescence associated genes: LIM Domain Protein (LIM) and *Arabidopsis *Aleurian-Like Proteinase (AALP, EC 3.4.22.-) were chosen from the literature [59,60] considering their role in drought-stress resistance and senescence, respectively. Finally, *in silico *survey of the *H. annuus *NCBI EST collection was performed using the stand alone version of SNP Discovery software [61] in order to identify putative polymorphisms. The software was able to assemble 6,972 contigs. Only alignments with the constraints of more than five members representing different germplasm sources, one or more SNPs detected and an associated function determined by BLASTx searches were considered (35 contigs). They were also analyzed to find ESTs members that correspond to the SSH-EST collection described by Fernández et al. [58] (31/35). Finally, 12 out of 31 candidate contigs from *in silico *survey were amplified for experimental validation. These sequences included: Ribosomal proteins L41 and S16 (RL41, RS16); enzymes such as S-Adenosylmethionine Decarboxilase (SAMC, EC 4.1.1.50), Sedoheptulose-1,7 Bisphosphatase Precursor (SBP, EC 3.1.3.37) and one Aminomethyltransferase (Glycine Cleavage System T Protein: GCvT, EC 2.1.2.10); a proteasome subunit (SEM); 3 chlorophyll binding proteins (Light Harvesting Chlorophyll A/B Binding Protein: LHCP; Chlorophyll A/B Binding Protein type III from the Photosystem I: PSI-III-CAB and Chlorophyll A/B Binding Protein: CAB); a Chloroplast Precursor from the Photosystem I (CPSI), a putative pathogenesis-related protein (Non-specific Lipid Transfer Protein: NsLTP) and one nucleotide transporter (Adenine Nucleotide Translocator: ANT). These regions are known to be involved in defense mechanisms against pathogens (NsLTP, SAMC), adaptation to various environmental stresses (RS16, CPSI, LHCP, CAB, PSI-III-CAB), regulation of Programmed Cell Death (RL41, ANT) and protein turnover pathways (SEM, GCvT) (Table 1).

Since patterns of polymorphism may differ greatly from locus to locus and thus, gene sampling may have a large impact on the levels of genetic diversity detected, Calmodulin (CAM), Chalcone Synthase (CHS; EC 2.3.1.74), Glyceraldehyde-3-Phosphate Dehydrogenase (GAPDH; EC 1.2.1.12), Cytosolic Phosphoglucose Isomerase (PGIC; EC 5.3.1.9), Gibberellic Acid Insensitive-Like Protein (GAI), Glutathione Peroxidase (GPX; EC 1.11.1.9), Glutathione S-Transferase (GST; EC 2.5.1.18) and Scarecrow-Like (SCR1 and SCR2) gene modulators previously used for the analyses of genetic diversity in sunflower [46] were also included for comparison purposes.

### Designing and testing of PCR primers

The tentative consensus (TC) from the DFCI *Helianthus annuus *Gene Index [62], with a given function associated by Blastx searches (probability threshold <1e-20), was used as template for primer design of the regions selected from literature and/or SSH-ESTs. Primer3 [63] was used for primers design. For the 9 genes: CAM, CHS, GAPDH, GPX, GST, PGIC, SCR1 and SCR2, the primers were synthesized either according to Liu and Burke [46] specification or re-designed with Primer3 software. The contigs from *in silico *analysis were amplified with primers designed over the longest EST within a contig, insuring the best probability to find most of the SNPs detected by the software.

Each PCR primer pair was used to amplify genomic DNA of HA89 and RHA266 for testing primer functionality. PCRs were performed in a 12 μl volume with 30 ng genomic DNA, 2 mM MgCl_2_, 0.2 mM dNTP, 1 U Taq Platinum Polymerase (Invitrogen, Buenos Aires, Argentina) and 0.25 mM primer set. The cycling conditions were: 2 min at 94° for initial denaturing, 35 cycles of 40 sec at 94°, 40 sec at 65–58°, 2 min 72°, and a final extension for 10 min at 72°. Amplified products were visualized under UV light after electrophoresis on an ethidium bromide-stained 1.0% agarose gel. Those primer sets that produced a single PCR product with both DNA genotypes were selected and amplified in the remaining 17 sunflower inbred lines using the conditions described above.

### Purification and sequence analysis of PCR products

The PCR fragments were prepared for sequence analysis by treating 10 μl of PCR reaction with 4 μl of EXOSAP-IT (Exonuclease I & Shrimp Alkaline Phosphatase, USB, Ohio, USA) or by QIAquick PCR Purification Kit (QIAGEN, Hilden, Germany). Those PCR products that could not be sequenced directly were cloned into pGEMT-easy (Promega, Madison, USA) and at least two clones were sequenced with forward and reverse primers to discard PCR errors.

The nucleotide sequences from both strands were obtained with an ABI 3130xl sequencer (Applied Biosystems, California, USA). When the credibility between the two reads was less than 98% a third sequencing assay was performed.

### SNP survey and analysis

ABI trace files were aligned using ABI Prism SeqScape Software version 2.5 (Applied Biosystems, California, USA). SeqScape quality values of base-calls were set ≥ 20, and default settings for the remaining parameters were used for SNPs and indel discovery. Polymorphisms which appeared only in one genotype were re-checked in chromatogram files. The coding and non-coding regions of each candidate gene were then identified by BLASTx searches.

The levels of genetic variation were estimated as nucleotide polymorphism (θ_W_[64]) and nucleotide diversity (π [65]). Watterson's θ is based on the number of segregating sites, while Tajima's π is based on the pairwise difference between sequences in the sample. To test the neutrality of mutations, we employed Tajima's D test [66] which is based on differences between π and θ. These parameters were obtained using the software package DnaSP 4.10.9 [67].

### Population structure and LD assessment

The analysis of population structure was performed with STRUCTURE 2.1 [52]. In this method, a number of clusters, groups or populations (K) are assumed to be present and to contribute to the genotypes of sampled individuals. Loci are assumed to be independent, and each K population is assumed to follow HWE. The number of groups evaluated ranged from 1 to 10. The analysis was performed using five replicate runs per K value, a burn-in period length of 200,000 and a run length of 10^*5*^. No prior information on the origin of individuals was used to define the groups. The allele frequencies were kept independent among clusters in order to avoid an overestimation of the number of clusters [68]. The run showing the highest posterior probability of data was considered for each K value.

Standardized disequilibrium coefficients (D') and squared allele-frequency correlations (r^*2*^) for pairs of loci are the preferred measures of LD. However, D' was not considered for the present analysis since it is strongly affected by small sample sizes, resulting in highly erratic behaviour when comparing loci with low allele frequencies [51]. Therefore, LD was measured using the r^*2 *^statistic obtained with DNAsp 4.10.9 [67]. The pairwise comparisons were pooled and plot together for the entire inbred lines set and also for one of the groups identified with STRUCTURE. Microcal™ Origin^*® *^Version: 7.5 (Microcal Software, Inc.) was used to fit the decay of r^*2 *^(pooled across loci).

## Abbreviations

SNP single nucleotide polymorphism, indels short insertions and/or deletions, SSRs simple sequence repeats, bp base pairs, kbp kilo base pairs, LD linkage disequilibrium, EST expressed sequence tags, SSH suppressed subtracted hybridization, HWE Hardy Weinberg equilibrium, IDs identification, P&I primitive and improved cultivated sunflowers, HD Homeo-Domain Proteins, ROS reactive oxygen species, PAs PolyAmines.

## Authors' contributions

CMF selected the candidate genes along with NBP. CMF amplified the regions and carried out SNPs and indel identification from the allele sequence data. CMF along with VVL performed the data analysis. RAH provided EST sequence information. NBP contributed to selection of germplasm. VVL highly assisted in the interpretation of the results. CMF, VVL and NBP wrote the manuscript. RAH and HEH helped to draft the manuscript. NBP and RAH conceived and coordinated the study. HEH initiated the project and contributed to the work by the interpretation and discussion of the data. All authors read and approved the manuscript.

## Supplementary Material

Additional file 1 **Candidate genes selected for SNP development and nucleotide diversity analysis**. The data displays the 64 candidate regions selected for SNP identification, the accession numbers of the sequences used for primer design and the putative functions associated by BLASTx searches, together with the protein accession best hit. Click here for file

## References

[B1] Oraguzie NC, Rikkerink EHA, Gardiner SE, De Silva HN, Oraguzie NC, Rikkerink EHA, Gardiner SE, De Silva HN (2007). Association Mapping in Plants.

[B2] Griffin TJ, Smith LM (2000). Single-nucleotide polymorphism analysis by MALDI-TOF mass spectrometry. Trends Biotechnol.

[B3] Schlotterer C (2004). The evolution of molecular markers--just a matter of fashion?. Nat Rev Genet.

[B4] Gupta PK, Roy JK, Prasad M (2001). Single nucleotide polymorphisms: A new paradigm for molecular marker technology and DNA polymorphism detection with emphasis on their use in plants. Curr Sci.

[B5] Kwok PY (2001). Methods for genotyping single nucleotide polymorphisms. Annu Rev Genomics Hum Genet.

[B6] Rafalski A (2002). Applications of single nucleotide polymorphisms in crop genetics. Curr Opin Plant Biol.

[B7] Fredman D, White SJ, Potter S, Eichler EE, Den Dunnen JT, Brookes AJ (2004). Complex SNP-related sequence variation in segmental genome duplications. Nat Genet.

[B8] Cho RJ, Mindrinos M, Richards DR, Sapolsky RJ, Anderson M, Drenkard E, Dewdney J, Reuber TL, Stammers M, Federspiel N, Theologis A, Yang WH, Hubbell E, Au M, Chung EY, Lashkari D, Lemieux B, Dean C, Lipshutz RJ, Ausubel FM, Davis RW, Oefner PJ (1999). Genome-wide mapping with biallelic markers in Arabidopsis thaliana. Nat Genet.

[B9] Cordeiro GM, Eliott F, McIntyre CL, Casu RE, Henry RJ (2006). Characterisation of single nucleotide polymorphisms in sugarcane ESTs. Theor Appl Genet.

[B10] Kota R, Rudd S, Facius A, Kolesov G, Thiel T, Zhang H, Stein N, Mayer K, Graner A (2003). Snipping polymorphisms from large EST collections in barley (Hordeum vulgare L.). Mol Genet Genomics.

[B11] Kota R, Varshney RK, Thiel T, Dehmer KJ, Graner A (2001). Generation and comparison of EST-derived SSRs and SNPs in barley (Hordeum vulgare L.). Hereditas.

[B12] Lai Z, Livingstone K, Zou Y, Church SA, Knapp SJ, Andrews J, Rieseberg LH (2005). Identification and mapping of SNPs from ESTs in sunflower. Theor Appl Genet.

[B13] Morales M, Roig E, Monforte AJ, Arus P, Garcia-Mas J (2004). Single-nucleotide polymorphisms detected in expressed sequence tags of melon (Cucumis melo L.). Genome.

[B14] Somers DJ, Kirkpatrick R, Moniwa M, Walsh A (2003). Mining single-nucleotide polymorphisms from hexaploid wheat ESTs. Genome.

[B15] Van K, Hwang EY, Kim MY, Kim IH, Cho YI, Cregan PB, Lee SH (2004). Discovery of single nucleotide polymorphisms in soybean using primers designed from ESTs. Euphytica.

[B16] Van K, Hwang EY, Kim MY, Park HJ, Lee SH, Cregan PB (2005). Discovery of SNPs in soybean genotypes frequently used as the parents of mapping populations in the United States and Korea. J Hered.

[B17] Zhu T, Salmeron J (2007). High-definition genome profiling for genetic marker discovery. Trends Plant Sci.

[B18] Feltus FA, Singh HP, Lohithaswa HC, Schulze SR, Silva TD, Paterson AH (2006). A comparative genomics strategy for targeted discovery of single-nucleotide polymorphisms and conserved-noncoding sequences in orphan crops. Plant Physiol.

[B19] Giancola S, McKhann HI, Berard A, Camilleri C, Durand S, Libeau P, Roux F, Reboud X, Gut IG, Brunel D (2006). Utilization of the three high-throughput SNP genotyping methods, the GOOD assay, Amplifluor and TaqMan, in diploid and polyploid plants. Theor Appl Genet.

[B20] Monna L, Ohta R, Masuda H, Koike A, Minobe Y (2006). Genome-wide searching of single-nucleotide polymorphisms among eight distantly and closely related rice cultivars (Oryza sativa L.) and a wild accession (Oryza rufipogon Griff.). DNA Res.

[B21] Rostoks N, Mudie S, Cardle L, Russell J, Ramsay L, Booth A, Svensson JT, Wanamaker SI, Walia H, Rodriguez EM, Hedley PE, Liu H, Morris J, Close TJ, Marshall DF, Waugh R (2005). Genome-wide SNP discovery and linkage analysis in barley based on genes responsive to abiotic stress. Mol Genet Genomics.

[B22] Schmid KJ, Sorensen TR, Stracke R, Torjek O, Altmann T, Mitchell-Olds T, Weisshaar B (2003). Large-scale identification and analysis of genome-wide single-nucleotide polymorphisms for mapping in Arabidopsis thaliana. Genome Res.

[B23] Caldwell KS, Russell J, Langridge P, Powell W (2006). Extreme population-dependent linkage disequilibrium detected in an inbreeding plant species, Hordeum vulgare. Genetics.

[B24] Ching A, Caldwell KS, Jung M, Dolan M, Smith OS, Tingey S, Morgante M, Rafalski AJ (2002). SNP frequency, haplotype structure and linkage disequilibrium in elite maize inbred lines. BMC Genetics.

[B25] Garris AJ, McCouch SR, Kresovich S (2003). Population structure and its effect on haplotype diversity and linkage disequilibrium surrounding the xa5 locus of rice (Oryza sativa L.). Genetics.

[B26] Nordborg M, Borevitz JO, Bergelson J, Berry CC, Chory J, Hagenblad J, Kreitman M, Maloof JN, Noyes T, Oefner PJ, Stahl EA, Weigel D (2002). The extent of linkage disequilibrium in Arabidopsis thaliana. Nat Genet.

[B27] Remington DL, Thornsberry JM, Matsuoka Y, Wilson LM, Whitt SR, Doebley J, Kresovich S, Goodman MM, Buckler ES (2001). Structure of linkage disequilibrium and phenotypic associations in the maize genome. Proc Natl Acad Sci USA.

[B28] Brown GR, Gill GP, Kuntz RJ, Langley CH, Neale DB (2004). Nucleotide diversity and linkage disequilibrium in loblolly pine. Proc Natl Acad Sci USA.

[B29] Ingvarsson PK (2005). Nucleotide polymorphism and linkage disequilibrium within and among natural populations of European aspen (Populus tremula L., Salicaceae). Genetics.

[B30] Auzanneau J, Huyghe C, Julier B, Barre P (2007). Linkage disequilibrium in synthetic varieties of perennial ryegrass. Theor Appl Genet.

[B31] Ponting RC, Drayton MC, Cogan NO, Dobrowolski MP, Spangenberg GC, Smith KF, Forster JW (2007). SNP discovery, validation, haplotype structure and linkage disequilibrium in full-length herbage nutritive quality genes of perennial ryegrass (Lolium perenne L.). Mol Genet Genomics.

[B32] Skot L, Humphreys J, Humphreys MO, Thorogood D, Gallagher J, Sanderson R, Armstead IP, Thomas ID (2007). Association of candidate genes with flowering time and water soluble carbohydrate content in Lolium perenne (L.). Genetics.

[B33] Xing Y, Frei U, Schejbel B, Asp T, Lubberstedt T (2007). Nucleotide diversity and linkage disequilibrium in 11 expressed resistance candidate genes in Lolium perenne. BMC Plant Biol.

[B34] Hamblin MT, Mitchell SE, White GM, Gallego J, Kukatla R, Wing RA, Paterson AH, Kresovich S (2004). Comparative population genetics of the panicoid grasses: sequence polymorphism, linkage disequilibrium and selection in a diverse sample of sorghum bicolor. Genetics.

[B35] Simko I, Haynes KG, Jones RW (2006). Assessment of linkage disequilibrium in potato genome with single nucleotide polymorphism markers. Genetics.

[B36] Somers DJ, Banks T, Depauw R, Fox S, Clarke J, Pozniak C, McCartney C (2007). Genome-wide linkage disequilibrium analysis in bread wheat and durum wheat. Genome.

[B37] Zhu YL, Song QJ, Hyten DL, Van Tassell CP, Matukumalli LK, Grimm DR, Hyatt SM, Fickus EW, Young ND, Cregan PB (2003). Single-nucleotide polymorphisms in soybean. Genetics.

[B38] Buckler EST, Thornsberry JM (2002). Plant molecular diversity and applications to genomics. Curr Opin Plant Biol.

[B39] Rieseberg LH, Seiler GJ (1990). Molecular evidence and the origin and development of the domesticated sunflower (Helianthus annuus, Asteraceae). Economical Botany.

[B40] Al-Chaarani GR, Gentzbittel L, Huang XQ, Sarrafi A (2004). Genotypic variation and identification of QTLs for agronomic traits, using AFLP and SSR markers in RILs of sunflower (Helianthus annuus L.). Theor Appl Genet.

[B41] Tang S, Knapp SJ (2003). Microsatellites uncover extraordinary diversity in native American land races and wild populations of cultivated sunflower.. Theor Appl Genet.

[B42] Paniego N, Echaide M, Munoz M, Fernandez L, Torales S, Faccio P, Fuxan I, Carrera M, Zandomeni R, Suarez EY, Hopp HE (2002). Microsatellite isolation and characterization in sunflower (Helianthus annuus L.). Genome.

[B43] Al-Chaarani GR, Roustaee A, Gentzbittel L, Mokrani L, Barrault G, champ-Guillaume G, Sarrafi A (2002). A QTL analysis of sunflower partial resistance to downy mildew ( Plasmopara halstedii) and black stem ( Phoma macdonaldii) by the use of recombinant inbred lines (RILs). Theor Appl Genet.

[B44] Poormohammad Kiani S, Talia P, Grieu P, Maury P, Hewezi T, Gentzbittel L, Sarrafi A (2007). Genetic analysis of plant water status and osmotic adjustment in recombinant inbred lines of sunflower under two water treatments. Plant Sci.

[B45] Tang S, Yu JK, Slabaugh B, Shintani K, Knapp J (2002). Simple sequence repeat map of the sunflower genome. Theor Appl Genet.

[B46] Liu A, Burke JM (2006). Patterns of nucleotide diversity in wild and cultivated sunflower. Genetics.

[B47] Kolkman JM, Berry ST, Leon A, Slabaugh MB, Tang S, Gao W, Shintani DK, Burke JM, Knapp SJ (2007). Single nucleotide polymorphisms and linkage disequilibrium in sunflower. Genetics.

[B48] Cheres MT, Knapp SJ (1998). Ancestral Origins and Genetic Diversity of Cultivated Sunflower: Coancestry Analysis of Public Germplasm. Crop Sci.

[B49] Roath WW, Miller JF, Gulya TJ (1981). Registration of RHA 801 sunflower germplasm. Crop Sci.

[B50] Zhang LS, Le Clerc V, Li S, Zhang D (2005). Establishment of an effective set of simple sequence repeat markers for sunflower variety identification and diversity assessment. Can J Bot.

[B51] Flint-Garcia SA, Thornsberry JM, Buckler ES (2003). Structure of linkage disequilibrium in plants. Annu Rev Plant Biol.

[B52] Pritchard JK, Stephens M, Donnelly P (2000). Inference of population structure using multilocus genotype data. Genetics.

[B53] Tenaillon MI, Sawkins MC, Long AD, Gaut RL, Doebley JF, Gaut BS (2001). Patterns of DNA sequence polymorphism along chromosome 1 of maize (Zea mays ssp. mays L.). Proc Natl Acad Sci USA.

[B54] Sato Y, Fukuda Y, Hirano HY (2001). Mutations that cause amino acid substitutions at the invariant positions in homeodomain of OSH3 KNOX protein suggest artificial selection during rice domestication. Genes Genet Syst.

[B55] Gaut BS, Long AD (2003). The lowdown on linkage disequilibrium. Plant Cell.

[B56] Flint-Garcia SA, Thuillet AC, Yu J, Pressoir G, Romero SM, Mitchell SE, Doebley J, Kresovich S, Goodman MM, Buckler ES (2005). Maize association population: a high-resolution platform for quantitative trait locus dissection. Plant J.

[B57] Gentzbittel LV, Bervillé A, Nicolas P (1995). Development of a consensus linkage RFLP map of cultivated sunflower (Helianthus annuus L.). Theor Appl Genet.

[B58] Fernandez P, Paniego N, Lew S, Hopp HE, Heinz RA (2003). Differential representation of sunflower ESTs in enriched organ-specific cDNA libraries in a small scale sequencing project. BMC Genomics.

[B59] Dezar CA, Gago GM, Gonzalez DH, Chan RL (2005). Hahb-4, a sunflower homeobox-leucine zipper gene, is a developmental regulator and confers drought tolerance to Arabidopsis thaliana plants. Transgenic Res.

[B60] Gepstein S, Sabehi G, Carp MJ, Hajouj T, Nesher MF, Yariv I, Dor C, Bassani M (2003). Large-scale identification of leaf senescence-associated genes. Plant J.

[B61] Barker G, Batley J, O' SH, Edwards KJ, Edwards D (2003). Redundancy based detection of sequence polymorphisms in expressed sequence tag data using autoSNP. Bioinformatics.

[B62] Dana Farber Cancer Institute TGIP The Helianthus annuus Gene Index (HaGI). http://compbio.dfci.harvard.edu/tgi/cgi-bin/tgi/gireport.pl?gudb=Sunflower.

[B63] Rozen S, Skaletsky H, Krawetz S, Misener S (2000). Primer3 on the WWW for general users and for biologist programmers. Bioinformatics Methods and Protocols: Methods in Molecular Biology.

[B64] Watterson GA (1975). On the number of segregating sites in genetical models without recombination. Theor Popul Biol.

[B65] Tajima F (1983). Evolutionary relationship of DNA sequences in finite populations. Genetics.

[B66] Tajima F (1989). Statistical method for testing the neutral mutation hypothesis by DNA polymorphism. Genetics.

[B67] Rozas J, Rozas R (1999). DnaSP version 3: an integrated program for molecular population genetics and molecular evolution analysis. Bioinformatics.

[B68] Falush D, Stephens M, Pritchard JK (2003). Inference of population structure using multilocus genotype data: linked loci and correlated allele frequencies. Genetics.

[B69] Fernandez P, Di Rienzo J, Fernandez L, Hopp HE, Paniego N, Heinz RA Transcriptomic identification of candidate genes involved in sunflower responses to chilling and salt stresses based on cDNA microarray analysis. BMC Plant Biol.

[B70] Bishop JG (2005). Directed mutagenesis confirms the functional importance of positively selected sites in polygalacturonase inhibitor protein. Mol Biol Evol.

[B71] Mallappa C, Yadav V, Negi P, Chattopadhyay S (2006). A basic leucine zipper transcription factor, G-box-binding factor 1, regulates blue light-mediated photomorphogenic growth in Arabidopsis. J Biol Chem.

[B72] Zimmermann G, Baumlein H, Mock HP, Himmelbach A, Schweizer P (2006). The multigene family encoding germin-like proteins of barley. Regulation and function in Basal host resistance. Plant Physiol.

[B73] Dezar CA, Tioni MF, Gonzalez DH, Chan RL (2003). Identification of three MADS-box genes expressed in sunflower capitulum. J Exp Bot.

[B74] Eason JR, Ryan DJ, Watson LM, Hedderley D, Christey MC, Braun RH, Coupe SA (2005). Suppression of the cysteine protease, aleurain, delays floret senescence in Brassica oleracea. Plant Mol Biol.

[B75] Arnaud D, Dejardin A, Leple JC, Lesage-Descauses MC, Pilate G (2007). Genome-wide analysis of LIM gene family in Populus trichocarpa, Arabidopsis thaliana, and Oryza sativa. DNA Res.

[B76] Kim MJ, Yoo YA, Kim HJ, Kang S, Kim YG, Kim JS, Yoo YD (2005). Mitochondrial ribosomal protein L41 mediates serum starvation-induced cell-cycle arrest through an increase of p21(WAF1/CIP1). Biochem Biophys Res Commun.

[B77] Woo HH, Hawes MC (1997). Cloning of genes whose expression is correlated with mitosis and localized in dividing cells in root caps of Pisum sativum L. Plant Mol Biol.

[B78] Lam E, Kato N, Lawton M (2001). Programmed cell death, mitochondria and the plant hypersensitive response. Nature.

[B79] Wood AJ, Joel Duff R, Oliver MJ (2000). The translational apparatus of Tortula ruralis: polysomal retention of transcripts encoding the ribosomal proteins RPS14, RPS16 and RPL23 in desiccated and rehydrated gametophytes. J Exp Bot.

[B80] Kader JC (1996). Lipid-Transfer Proteins in Plants. Annu Rev Plant Physiol Plant Mol Biol.

[B81] Smalle J, Vierstra RD (2004). The ubiquitin 26S proteasome proteolytic pathway. Annu Rev Plant Biol.

[B82] Wi SJ, Kim WT, Park KY (2006). Overexpression of carnation S-adenosylmethionine decarboxylase gene generates a broad-spectrum tolerance to abiotic stresses in transgenic tobacco plants. Plant Cell Rep.

[B83] Lee HH, Kim DJ, Ahn HJ, Ha JY, Suh SW (2004). Crystal structure of T-protein of the glycine cleavage system. Cofactor binding, insights into H-protein recognition, and molecular basis for understanding nonketotic hyperglycinemia. J Biol Chem.

[B84] Lefebvre S, Lawson T, Zakhleniuk OV, Lloyd JC, Raines CA, Fryer M (2005). Increased sedoheptulose-1,7-bisphosphatase activity in transgenic tobacco plants stimulates photosynthesis and growth from an early stage in development. Plant Physiol.

[B85] Coberly LC, Rausher MD (2003). Analysis of a chalcone synthase mutant in Ipomoea purpurea reveals a novel function for flavonoids: amelioration of heat stress. Mol Ecol.

[B86] Savenstrand H, Brosche M, Strid A (2004). Ultraviolet-B signalling: Arabidopsis brassinosteroid mutants are defective in UV-B regulated defence gene expression. Plant Physiol Biochem.

